# Alzheimer’s disease: amyloid-based pathogenesis and potential therapies

**DOI:** 10.15698/cst2018.07.143

**Published:** 2018-06-29

**Authors:** Yixiu Zhou, Yuhui Sun, Quan-Hong Ma, Yaobo Liu

**Affiliations:** 1 Medical College of Soochow University, Soochow University, Suzhou, Jiangsu 215004, China.; 2 Jiangsu Key Laboratory of Neuropsychiatric Diseases and Institute of Neuroscience, Soochow University, Suzhou, Jiangsu 215123, China.

**Keywords:** Alzheimer’s disease (AD), amyloid β (Aβ), synapses, neural circuits, treatment

## Abstract

Alzheimer’s disease is one of the most severe neurodegenerative diseases among elderly people. Different pathogenic factors for Alzheimer’s disease have been posited and studied in recent decades, but no effective treatment has been found, necessitating further studies. In this Viewpoint article, we assess studies on the mechanisms underlying the accumulation of amyloid (Aβ) peptide and the formation of Aβ oligomers because their accumulation in amyloid plaques in brain tissue has become a well-studied hallmark of Alzheimer’s disease. We focus on the production of Aβ and its impact on the function of synapses and neural circuits, and also discuss the clinical prospects for amyloid-targeted therapies.

## INTRODUCTION

Alzheimer’s disease (AD) is one of the most prevalent forms of dementia; in 2015, for example, AD affected ~46.8 million people worldwide. It is estimated that this number will continue to increase and reach 131.5 million in 2050 1. In China in 2014, for instance**,** ~10% of the population was 60 years of age or older (~212 million people), according to the National Bureau of Statistics of China 2. Recent increase in life expectancy may greatly expand the future AD burden 3, and indeed, AD will have a larger impact on the economy of China and of the world 3. Thus, a great deal of effort has been spent on studying the pathological mechanism of AD and on trying to find a treatment to cure AD. In this Viewpoint, we discuss one of the most central hypotheses, namely the amyloid cascade hypothesis, and subsequent research that complements or challenges it. A fundamental aspect of research on AD concerns the involvement of amyloid β (Aβ) in the pathophysiology of the disease and as a possible target for treatment. Despite these efforts, however, no anti-amyloid therapy has yet been established 4. Hence, this Viewpoint focuses on attempts to research on amyloid-based pathogenesis and develop an amyloid-targeted therapy.

## THE AMYLOID CASCADE HYPOTHESIS AND SUBSEQUENT STUDIES

One of the characteristic pathologies of AD is the presence of parenchymal amyloid plaques in the brain tissue of patients 5. Aβ was first isolated from the meningeal vessels of AD patients in 1984 6. One year later, the same peptide was identified as the core of senile plaques observed in the brain tissue of AD patients 7. These findings called researchers’ attention to the accumulation of the amyloid protein. Moreover, it was discovered that Down syndrome (trisomy 21) patients often develop AD later in life and the amyloid precursor protein (APP) gene is located on chromosome 21 8. Thus, the amyloid cascade hypothesis was first posited in 1992, which postulates that the accumulation of Aβ peptides initiates the pathogenesis of AD, leading to neurofibrillary tangles and neurodegeneration that cause memory loss 9. Hardy *et al*. proposed that the overproduction of Aβ results from hyperactivation of the β and γ secretases (gain-of-function mechanism), which cleave APP and yield Aβ 8. In the years since the hypothesis was proposed, the Aβ peptide has been a star molecule in most of the research on the pathophysiology of AD.

The amyloid cascade hypothesis has generated a lively discussion whether plaques are neurotoxic or protective. Although it was previously believed that plaques are the initiators of disease pathogenesis, Lee *et al*. argued that all available data are also consistent with the conclusion that amyloid plaques actually constitute a protective adaptation 10. Meanwhile, Bishop *et al*. found that this apparent paradox became evident when Aβ was bound to metal ions, and the resulting complex could be neurotoxic or neuroprotective 11. Moreover, it has been reported that soluble Aβ oligomers can impair synapse structure and function and that the smallest synaptotoxic species are dimers, whereas neither Aβ monomers nor soluble amyloid plaque cores significantly alter synaptic plasticity 12. Now it is generally agreed that the soluble Aβ oligomers, rather than amyloid plaques, are synaptotoxic.

Aside from the debate concerning amyloid plaques and oligomers, new findings have arisen supporting the amyloid cascade hypothesis. Jonsson *et al*. made an astonishing discovery that a coding mutation (A673T) in the APP gene could protect against AD and cognitive decline in an elderly population with AD, which indicated that a reduction of (-cleavage of APP might protect against AD 13. He *et al*. found that Aβ enhanced tau pathogenesis by creating a unique environment that facilitated tau aggregation at an early stage and helped translocate the tau "seeds" at a later stage 14.

However, although the accumulation of Aβ is acknowledged as a key factor in the cognitive deficit observed in AD patients, other studies have pointed out the weakness of the original amyloid cascade hypothesis and pointed out some challenges. Researchers raised concerns about the amyloid cascade hypothesis based on studies of familial AD (FAD), which is attributable to mutations in one of three genes, namely presenilin1 (PSEN1), presenilin2 (PSEN2), or APP 15. Among them, presenilin1 and presenilin2 are the core components of γ-secretase, which cleaves the C-terminal fragment of APP produced by β-secretase cleavage within the plasma membrane, releasing Aβ 16. Thus, many studies on FAD have focused on mutations in the gene encoding γ-secretase. For instance, Xia *et al*. suggested that PSEN1 gene mutations could both abolish γ-secretase activity, which decreases the production of Aβ42 and Aβ40, and increase the Aβ42/Aβ40 ratio, which promotes Aβ deposition through a loss-of-function mechanism linked to familial AD onset 17. Ben-Gedalya *et al*. reported that inhibition of cyclophilin B leads to presenilin1 misfolding, aggregation, and deposition, which reduces γ-secretase function and thus opposes the gain-of-function mechanism 18. However, Szaruga *et al*. expressed doubt about the loss-of-function hypothesis and proposed an alternative view that "*pathogenic mutations in PSEN cause disease by qualitative shifts in Aβ profile production (γ-secretase dysfunction)*" 19. In response to these challenges, Hardy *et al*. later argued that the loss-of-function hypothesis might overlook the elevation of Aβ43 and of other, longer Aβ species 20.

Moreover, some researchers have suggested that the simple linear pathway of tracing disease progression from Aβ to AD should be rejected 21. Some clinical studies have reported that cognitive decline correlates only weakly with changes in Aβ burden and that a window of time exists between Aβ accumulation and AD onset 22. Moreover, although the antineoplastic drug bexarotene rapidly clears amyloid plaques in mouse brain and reverses the cognitive decline of the mice 23, clinical trials with humans have not proved promising. For instance, the drug AN1792 could eliminate amyloid plaques quite well, but it could not reverse the neurodegeneration 24. Thus, the opinion that the amyloid cascade hypothesis should be rejected is based on the fact that Aβ accumulation does not correlate with the immediate onset of AD and that elimination of amyloid plaques cannot stop neurodegeneration. However, because Aβ plaques are not necessarily sources of toxicity, as mentioned above, more evidence may be needed before the amyloid cascade hypothesis can be soundly rejected.

Also, compared with early-onset FAD, late-onset/sporadic AD, which affects most AD patients, has shown a different mechanism for pathogenesis. Indeed, most sporadic AD cases display normal γ-secretase activity, in contrast to FAD 19. Moreover, neurofibrillary tangles develop sooner in PSEN-FAD, portending more rapid neuronal demise 25. However, Thomas *et al*. suggested that changes in functional connectivity manifest similarly in both types of AD, and therefore early-onset AD might serve as a model for late-onset AD studies 26.

Thus, there are different and competing views regarding the amyloid cascade hypothesis, and no definite conclusions can be drawn at this time.

## PRODUCTION OF Aβ

The Aβ peptides are proteolytic fragments derived from APP, which is an integral membrane protein found to exhibit both neurotoxic and neurotrophic protective effects 16. The human APP gene is located on the long arm of the chromosome 21, and alternative splicing can produce various APP mRNAs encoding several isoforms 27. The most common APP species in the brain is APP695, and it is produced mainly by neurons 28. APP is synthesized and transported to the plasma membrane via the endoplasmic reticulum-Golgi secretory pathway 29. Also, it has been proposed that APP can function as a cell-surface receptor, which can be bound by Aβ and regulate the production and downstream signaling of Aβ 27. APP is transported along axons to presynaptic terminals, where it accumulates and leads to Aβ deposition at synapses 28. Notably, two pathways are known to process APP, namely the amyloidogenic and non-amyloidogenic pathways 16, and the latter is the principal pathway under physiological conditions 30.

**Figure 1:**
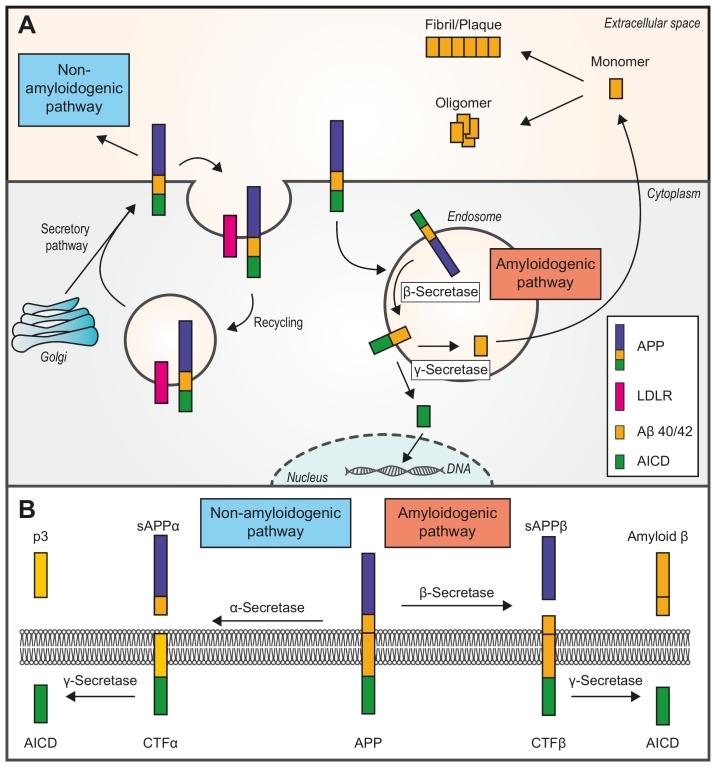
FIGURE 1: Two pathways of amyloid β (Aβ) peptides generation. Amyloid precursor protein (APP) is a type I integral membrane protein that is cleaved at different sites to yield various products. **(A)** An overview of the two pathways. APP is translocated from the cytoplasm into the endoplasmic reticulum where it enters the secretory pathway and then is transported to the neuronal plasma membrane. The majority of APP is processed via the non-amyloidogenic pathway (see panel B). APP can be recycled in endosomes by binding to LDLRs (low-density lipoprotein receptors). The right side of the figure shows the amyloidogenic pathway. Unlike the non-amyloidogenic pathway, which is carried out at the plasma membrane, the amyloidogenic pathway mainly occurs in endosomes. Ultimately, AICD is transferred to the nucleus, where it functions as a transcriptional factor, whereas the Aβ40/42 monomer is removed to the extracellular space. Monomers aggregate either into oligomers or fibrils/plaques. **(B)** Non-amyloidogenic (left) or amyloidogenic (right) pathway of APP processing. In the non-amyloidogenic pathway, APP is first cleaved by α-secretase, releasing a soluble ectodomain of APP called sAPPα and a membrane-tethered intracellular C-terminal fragment (CTFα). Then, the C-terminal fragment is further cleaved by γ-secretase to produce a 3-kDa peptide (p3) and an APP intracellular domain (AICD). In the amyloidogenic pathway, the products of β-secretase are a soluble ectodomain of APP (sAPPβ) and a C-terminal fragment β (CTFβ). The second step releases Amyloid ( and AICD. *APP, amyloid precursor protein. CTF alpha/beta, C-terminal fragment alpha/beta. sAPP alpha/beta, soluble ectodomain of APP. p3, 3-kD peptide. AICD, APP intracellular domain.*

In the non-amyloidogenic pathway (**Fig. 1A** and **Fig. 1B**), APP is first cleaved by α-secretase, which is a member of the ADAM (A Disintegrin And Metalloproteinase) family and is abundant at the plasma membrane. This cleavage yields the soluble ectodomain sAPPα and leaves the C-terminal fragment alpha (CTFα) in the plasma membrane. Subsequent cleavage of CTFα by γ-secretase releases a soluble extracellular peptide (p3) and the APP intracellular domain (AICD) 16. Of note, the APP holoprotein can be bound by various low-density lipoprotein receptors (LDLRs), such as SORL1, which is an APP-specific sorting receptor. Any APP that binds LDLRs can be internalized and enters a recycling pathway. The absence of LDLRs can shunt APP into the β-secretase cleavage pathway (the amyloidogenic pathway) 31.

The amyloidogenic pathway (**Fig. 1A** and **Fig. 1B**) involves APP trafficking through the secretory and recycling pathways where APP interacts with β- and γ-secretases 32. After APP is internalized and delivered to endosomes 30, the first step of the pathway is catalyzed by β-secretase 1 (BACE-1), a single-transmembrane aspartyl protease. BACE-1 cleaves APP and generates the soluble ectodomain sAPPβ and CTFβ (left in the membrane). The subsequent hydrolysis of CTFβ by γ-secretase yields AICDs and Aβ monomers, which have dual physiological effects 16. The majority of Aβ peptides are secreted to the extracellular space, although a small amount can aggregate inside neurons 30, whereas AICD is transported into the nucleus where it functions as a transcription factor 29. In addition, γ-secretase cleaves CTFβ at different sites and in multiple sequential steps, which ultimately produces mainly two species of Aβ, namely Aβ40 and Aβ42 27. Under basal conditions, Aβ40 comprises ~90% of all Aβ produced 16.

The fact that various Aβ species are produced has a pathogenic impact on neurons, and the longer Aβ42 is believed to be more toxic than Aβ40 33. In addition, it has been reported that the ratio of Aβ42 to Aβ40 might predict the severity of AD 34, and indeed, this ratio has long been used as a biomarker in AD research. Early studies reported that PSEN mutations increase the Aβ42/Aβ40 ratio 17. In the amyloidogenic pathway, γ-secretase can trim the epsilon site and gamma site of the transmembrane CTFβ. Thus, both mutations in the domain of the epsilon/gamma sites and the γ-secretase modulators can influence Aβ42 production. Mutations in the transmembrane domain of APP can increase the Aβ42/Aβ40 ratio, leading to aggressive early-onset FAD, whereas γ-secretase modulators can decrease the level of Aβ42 and thus have therapeutic potential 35. In a recent investigation, Johnson *et al*. proposed that, under physiological conditions, small Aβ oligomers bound to the plasma membrane and further oligomerized with kinetics depending on the local Aβ42/Aβ40 ratio 36. Siegel *et al*. discovered that the ratio was greatest for Aβ’ (the N-terminally truncated Aβ_11-x _produced from the 89-residue CTF), followed by Aβ and then p3, which provided new insight for the development of γ-secretase modulators 37. Moreover, others suggested that the ratio could be used as the biomarker for the diagnosis of neurochemical dementia 38,39 as well as in clinical trials targeting cognitively normal individuals with high brain Aβ levels 40.

Once the Aβ monomer is produced, it either goes through the degradation process or accumulates to form other species of amyloids, such as oligomers, fibrils, etc. There are two main mechanisms by which amyloids are removed from cells. First, the monomer that forms in endosomes can be transferred to lysosomes in the neuron, where it is degraded. Second, if the monomer is released to the outside of the neuron, microglia can destroy it by releasing insulin-degrading enzyme 29. Aside from this, there are two models for the formation of Aβ fibrillogenesis. The classic model posits that fibril formation is a nucleation-dependent polymerization process in which monomers give rise to oligomers, from which protofibrils form. Subsequently the protofibrils emanate full-length fibers. The new model, however, implies that protofibrils cannot form fibrils directly. Instead, protofibrils may be the precursors for fibrillogenesis 41. Also, after studying several species of amyloids, i.e., monomers, oligomers, Aβ*56, etc., Ono noted that "*soluble pre-fibrillar aggregates, that is, oligomers of Aβ, are proximate neurotoxins*" 41.

Compared with Aβ42, which has been studied for many years, Aβ43 has been quite overlooked. A study published in 2011, however, suggested that Aβ43 is potentially toxic and amyloidogenic, perhaps to an extent greater than Aβ42 42.

A new APP processing pathway was recently identified. This pathway generates proteolytic fragments of APP capable of inhibiting neuronal activity within the hippocampus. The cleavage of APP by η-secretase yields CTFη, which is cleaved by ADAM10 and BACE1 into long and short amyloid eta (Aηα, Aηβ). CTFη is abundant in dystrophic neurites, and its generation is usually mediated by membrane-bound matrix metalloproteinase 43. Also, Aβ42 disrupts the barrier between the blood and cerebrospinal fluid via activation of matrix metalloproteinase 44. Thus, a connection may exist between these two pathways.

## Aβ AFFECTS SYNAPTIC AND NEURAL CIRCUIT FUNCTION

As mentioned above, Aβ peptide plays an important role in AD by influencing synapses and, in turn, neural circuits. Accumulation of Aβ in the brain parenchyma can lead to loss of dendritic spines and synapses as well as alterations in synaptic transmission and neural activity 45.

Although overproduction of the Aβ oligomer is pathogenic, a normal level of Aβ helps to maintain physiological homeostasis. A study using mouse/rat hippocampal slices suggested that Aβ might have a normal negative feedback function that regulates APP processing; in that study, Kamenetz *et al*. proposed that an elevated level of neuronal activity could enhance the activity of β-secretase, which would generate Aβ monomers. Overproduction of these monomers leads to synaptic depression, which in turn suppresses neuronal activity and further reduces Aβ production 46. This constitutes a protective mechanism that modulates the production of Aβ monomers and can prevent monomer overproduction in nearby neurons. Nevertheless, a study using APP-transfected neurons in rat hippocampal slice cultures revealed that structural plasticity was reversibly impaired in APP-overexpressing cells 47. Continuous overproduction of Aβ oligomers at either dendrites or axons can lower spine density and plasticity; this "synaptic pruning" in spine number can be reduced by blocking action potentials, nicotinic acetylcholine receptors (nAChRs), or N-methyl-d-aspartic acid (NMDA) receptors (NMDARs) 47. Aβ-mediated spine loss requires the upregulation of NMDA-type glutamate receptor-dependent activity and the subsequent cascade of signaling that includes cofilin and calcineurin. Shankar *et al*. experimented on mouse hippocampal neurons and found that activation of NMDARs could induce either long-term depression (LTD) or long-term potentiation (LTP) depending on the Ca^2+^ concentration. Soluble Aβ oligomers can partially block NMDARs, which either reduces Ca^2+^ influx or enhances NMDAR-dependent activation of calcineurin. Decreased Ca^2+^ influx through NMDARs can induce LTD through a calcineurin-dependent pathway 48. Also, GSK3 activity stimulated by Aβ may lead to NMDAR-dependent LTD and inhibit LTP 49, resulting in loss of synaptic spine and eventually neurodegeneration 50.

Because the dendritic spine is part of a synapse, spine loss can also affect synaptic activity. It has been suggested that Aβ oligomers play a central role in controlling neural activity at specific types of synapses which affects the neural circuits 51. Aside from NMDARs, Aβ oligomers are heterogeneous and have high affinity to specific types of receptors, which activate various signaling pathways leading to inevitable cell death 52. For instance, Lauren *et al*. identified the cellular prion protein (PrP^C^) as an Aβ oligomer receptor by expression cloning and discovered that PrP^C^ mediated the inhibition of LTP in a wild-type mouse hippocampal slice by the binding of Aβ42 oligomers. Presumably, PrP^C^ interacts with NMDAR subunit 2D and inhibits its function by initiating a signaling cascade that modifies synaptic morphology and functions in the brain 53. Kim *et al*. observed that murine PirB (paired immunoglobulin-like receptor B) was associated with memory deficits in adult mice as well as loss of synaptic plasticity in the juvenile visual cortex. The selective binding of Aβ42 to PirB could lead to increased interactions between PirB and cofilin or protein phosphatases in APP/presenilin1 mice. They proposed that the human homolog LilrB2 (leukocyte immunoglobulin-like receptor B2) might also enhance cofilin signaling, which is seen in the human AD brain 54. Yamamoto *et al*. suggested that Aβ oligomers can lead to nerve growth factor receptor-mediated cell death through the p75 neurotrophin receptor 55. Zhao *et al*. reported that Aβ oligomers can impair the function of neuronal insulin receptors in rat hippocampal and cortical neurons, indicating that insulin resistance in the AD brain is a response to the Aβ-derived diffusible ligands (ADDLs) 56. Moreover, insulin receptor-mediated interference with Aβ production prevented the rapid activation of a specific kinase required for LTP 57. Also, Magdesian *et al*. found that Aβ oligomers could bind to the Frizzled cysteine-rich domain at, or in close proximity to, the Wnt-binding site and inhibit the canonical Wnt signaling pathway, which caused tau phosphorylation and neurofibrillary tangles 58. Moreover, it has even been reported that Aβ peptides can form oligomeric ion channels, assisted by cholesterol, and that these channels can induce an increase of Ca^2+^ level in neurons 59. Furthermore, coupled with the increase in membrane permeability induced by Aβ oligomers 60, the channels can disrupt Ca^2+^ homeostasis and lead to neurodegeneration.

**Figure 2:**
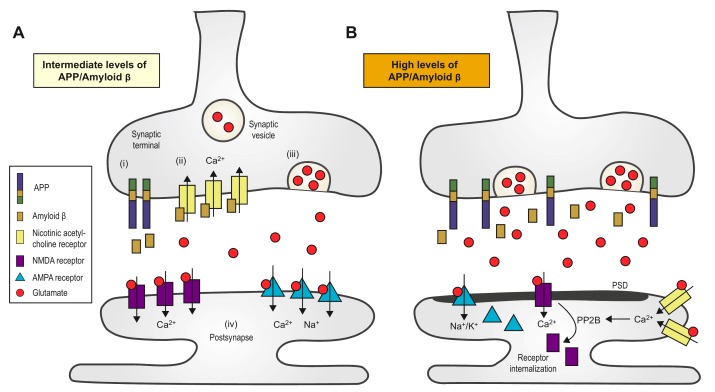
FIGURE 2: Synaptic transmission regulated by amyloid ( (Aβ). **(A)** An intermediate increase in Aβ level can only increase the probability of releasing synaptic vesicles. The processing of amyloid precursor protein (APP) at the synaptic terminal yields Aβ peptides (i), which bind to and activate the presynaptic (7-nAChRs (ii). Moreover, the subsequent influx of Ca^2+^ is mediated by nAChRs, which, in turn, trigger the release of glutamate from the synaptic vesicles (iii). Glutamate activates both AMPARs and NMDARs, which induce synaptic potentiation (iv). **(B)** A dramatic increase of Aβ, however, can lead to LTD. First, accumulation of glutamate results in the long-term activation of NMDARs and AMPARs, which facilitates their internalization. Second, Aβ might redistribute NMDARs. Third, the activation of perisynaptic (7-nAChRs activates the protein phosphatase 2B (PP2B), a Ca^2+^-sensitive enzyme that induces the internalization of NMDARs 67. *(7-nAChR, (7-nicotinic acetylcholine receptor.* *PSD, post-synaptic density*. *NMDAR, N-methyl-D-aspartate receptor. AMPAR, α-amino-3-hydroxy-5-methyl-4-isoxazolepropionic acid receptor.*

Normally, a small increase in Aβ level will increase the probability of releasing synaptic vesicles (**Fig. 2A**). Such an increase can promote activation of presynaptic acetylcholine receptors, which increases the internal concentration of Ca^2+^. The high concentration of Ca^2+^ increases glutamate release and promotes excitatory neural activity 51,61. Aβ can participate in a positive feedback loop in which it binds to nAChRs that are close to the site of Aβ secretion, resulting in increased intracellular Ca^2+^. An increase in Ca^2+^ concentration may increase Aβ production 47. It is possible that an excessive increase of Aβ will lead to the aforementioned negative feedback loop and eventually to compensatory suppression of the neuronal activity 46. Palop *et al*. suggested that Aβ can trigger intermittent and aberrant excitatory neuronal activity in the cortex and hippocampus, which might result in remodeling of the inhibitory circuitry. They proposed that a "high level of Aβ induces aberrant excitatory neuronal activity, which triggers compensatory inhibitory mechanisms to counteract overexcitation", and both the excitation and inhibition might be involved in AD-related network dysfunction 62. A high level of Aβ leads to aberrant neuronal activity by enhancing synchrony among the remaining glutamatergic synapses 51. The acute effects induced by endogenous Aβ in certain studies were exclusively presynaptic. An increase or significant decrease in Aβ level impairs short-term synaptic facilitation 61. Thus, the level of Aβ must be maintained within an intermediate range, and either a low or high level can negatively impact presynaptic facilitation by decreasing presynaptic efficacy or postsynaptic depression. It has been suggested that excitatory synapses are highly sensitive to changes in Aβ level, whereas inhibitory synapses are relatively immune to the immediate effects of Aβ 61. The relative decrease in inhibition can lead to hyperactivity as well as abnormal synchronization 63. In addition, Siskova *et al*. reported that interneurons can alter their excitability and synchronizing function owing to cell type-specific vulnerability and/or persistently altered input. Thus, dendritic structural dysfunctions may be linked to neuronal hyperexcitability 64.

A small increase in Aβ will enhance LTP and memory 61, whereas an acute increase in synaptic Aβ can induce LTD instead 51,65. A decrease in the density of postsynaptic glutamate receptors, such as AMPARs and NMDARs, and the activation of the calcineurin-dependent pathway, which is involved in Aβ-induced spine loss, is also necessary for LTD 49. Accumulation of glutamate initially results in postsynaptic depolarization through AMPARs and then the activation of NMDARs 66. However, long-term activation leads to receptor desensitization and internalization of NMDARs and AMPARs. Moreover, the binding and activation of Aβ on alpha-7 nAChRs induces endocytosis of NMDARs through the action of protein phosphatase 2B 67. Changes in the number of the NMDARs can affect NMDAR-dependent Ca^2+^ influx, which is responsible for initiating LTD (**Fig. 2B**). Activation of perisynaptic NMDARs can lead to LTD 66, and over-activation of extrasynaptic NR2B-containing NMDARs can inhibit hippocampal LTP. Soluble Aβ increases the phosphorylation of p38 MAPK, which contributes to the inhibition of LTP and subsequently impairs the ERK and CREB signaling pathway 68. Um *et al*. demonstrated that Aβo/PrP^C ^complexes could activate the Fyn signaling pathway, which increases the density of cell-surface NMDARs and excitotoxicity, resulting in the loss of both dendritic spines and cell-surface receptors 69.

It has been suggested that the formation of neural circuits and memories is impaired by weakened connectivity owing to chronic elevation of Aβ 47. An increased level of Aβ results in an aberrant excitatory network and compensatory inhibition of learning and memory circuits, which promotes cognitive decline 62. Long-term accumulation of Aβ, disinhibition of excitatory cells, and synaptic loss lead to neuronal hyperactivity, which may lead to epileptiform activity 45,70. Coincidently, in some pre-symptomatic individuals who eventually develop AD, neuronal hyperactivity was found in regions - such as hippocampus - that are associated with learning and memory 45. Additionally, AD impairs slow wave oscillations, which consolidate recently acquired memories in the cortical area, thalamus, and hippocampus 63. Moreover, excessive oligomeric Aβ binding to cell-surface receptors can induce neuronal apoptosis 52. Long-term accumulation of Aβ results in oxidative damage to both DNA and proteins, which also leads to cell death 71 and impairs the affected brain regions, although the level of Aβ plateaus before the onset of rapid neurodegeneration and cognitive symptoms 72. Thus, excessive neuronal activity, hyper-synchrony, impaired oscillations, and cell death, etc., could be key features of AD. It should be noted that aside from Aβ, GABAergic dysfunction also contributes to the formation of the aberrant neural networks that are typical of AD 51. Thus, diverse mechanisms may contribute to neural network dysfunction in AD.

## TREATMENT BASED ON Aβ HAS NOT BEEN ENTIRELY SUCCESSFUL

**Figure 3:**
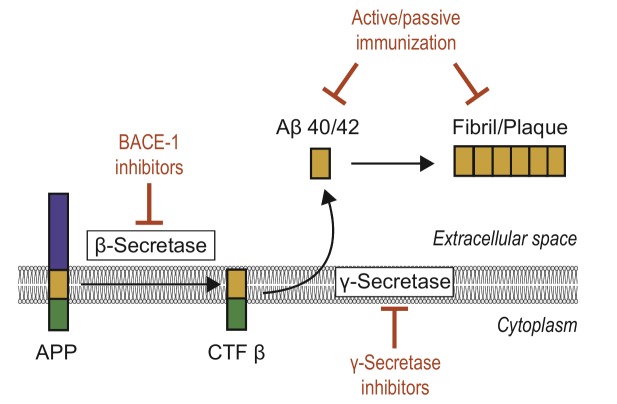
FIGURE 3: Current treatment strategies based on the amyloid cascade hypothesis. Recent or ongoing clinical trials have mainly focused on inhibition of β-secretase (BACE-1), γ-secretase, or immunization strategies against Aβ monomers or plaques. See text for details. *CTFβ, C-terminal fragment beta.*

Owing to its key role in AD, amyloid-targeted therapy has become a major research interest. There are two main ways to reduce excessive level of Aβ in neurons, namely (i) to correct the aberrant generation of Aβ and (ii) repair the faulty clearance mechanism (**Fig. 3**).

One attractive therapeutic strategy to reduce Aβ production are drugs that modulate the activity of the enzymes β-secretase or γ-secretase, especially inhibitors of β-secretase (BACE-1). Although considerable efforts have been made to develop BACE-1 inhibitors, most trials have failed due to insufficient target specificity, brain permeability, and/or research design without testing cognitive outcomes or measuring Aβ level 71,73. The first generation of large-molecule drugs failed because of their unfavorable pharmacological properties, such as the inability to cross the blood-brain barrier (BBB), and the second generation of small-molecule compounds still could not effectively penetrate the BBB 71. The third-generation drugs, such as verubecestat (Merck & Co.), AZD-3293 (AstraZeneca and Eli Lilly), and JNJ-54861911 (Janssen Research & Development), are BACE-1 inhibitors currently in Phase III trials 74. In February 2017, Merck halted its late-stage trial of verubecestat for mild-to-moderate Alzheimer's disease (EPOCH) after it was reported as having "*virtually no chance of finding a positive clinical effect*" according to an independent panel of experts 75. With respect to patients with prodromal Alzheimer's disease (APECS), however, the results of Merck's trial of verubecestat for that purpose are expected in February 2019 75. However, AZD-3293 yielded favorable results in Phase I; this compound can cross the BBB and is orally active and well tolerated up to the highest dose given. In September 2014, a large, pivotal phase II/III trial (AMARANTH) started and another Phase III trial (DAYBREAK-ALZ) was initiated in 2016 for AD patients with mild dementia. These trials will end in 2019 and 2021, respectively 76. As for γ-secretase, the failure to develop an inhibitor is attributable to a limited understanding of the structure of γ-secretase. Inhibitors like semagacestat proved ineffective in clinical trials 73. Although previous results were not satisfying, peptide-based aggregation inhibitors hold significant promise for future AD therapy owing to their high selectivity and low toxicity, among other attributes 77.

Alternatively, both active and passive strategies of immunization with the peptide are potential routes to enhance amyloid plaque clearance in the parenchyma. Active immunization is achieved via immunization with intact Aβ42 peptide or Aβ fragments, whereas passive immunization is achieved with anti-Aβ antibodies 78.

Some of the trials that have focused on active immunization with Aβ succeeded in reducing the level of Aβ peptide in patients, but with severe side effects, such as subacute meningoencephalitis (AN1792, Janssen, Pfizer) 79. Although the trials were halted, subsequent investigations were carried with former participants. Holmes *et al*. reported that AN1792 could eliminate amyloid plaques fairly well, but the treatment did not stop the process of neurodegeneration 24, which lends credence to the opinion that amyloid plaques are not necessarily neurotoxic. Another study in 2015 showed that AN1792 could accelerate the removal of damaged neurons involving activated microglia 80. Aside from AN1792, investigators have also developed other active immunization strategies. For instance, Mulder *et al*. demonstrated that a trivalent vaccine of small Aβ-derived cyclopeptide conjugates could effectively induce a specific antibody response against misfolded Aβ without noticeable side effects 81. Further, a trial with CAD106 (Novartis Pharmaceuticals Corporation) is now in Phase II/III after five multicenter Phase II trials. Unlike AN1792, CAD106 is generally well tolerated, with no evidence of central nervous system inflammation 82. Recently, a P-particle-based Aβ epitope vaccine showed promise by reducing amyloid deposition, rescuing memory loss, and restoring Aβ homeostasis in vivo 83. Thus, active immunization might still be a potential therapy for AD.

In recent years, research interests have focused on developing monoclonal antibodies against Aβ; for example, solanezumab against the middle region of Aβ (developed by Ely Lilly), aducanumab against aggregated Aβ (tested by Biogen), and bapineuzumab against the N-terminus of Aβ (directed by OOP & Johnson), among others. The efficacy of Aβ immunization strategies has not been consistent across clinical trials 84. Although trials of Aβ monoclonal antibodies such as solanezumab and bapineuzumab have proved effective for reducing Aβ level, little improvement in cognition has been achieved for AD patients. In two Phase III clinical trials in 2012, solanezumab, which was once considered the most promising drug for AD treatment, failed to show significant cognition benefits. In a subsequent Phase III trial that ended in November 2016, solanezumab did not show a significant effect on slowing cognitive decline in patients mildly affected with AD 85. Recently, a more exciting Phase Ib study showed that treatment with aducanumab (BIIB037), a human monoclonal antibody against aggregated Aβ, reduced Aβ deposits in the brain in a dose- and time-dependent manner, as assessed with florbetapir-based PET imaging. This suggested a slowing of clinical progression based on scores from the Clinical Dementia Rating-Sum of Boxes and Mini Mental State Examination 86.

All in all, the failure of numerous clinical trials of anti-amyloid agents can be attributed to many factors, such as inadequate preclinical data, poor brain penetration, or poor understanding of amyloid function, among others. In a review, Hardy *et al*. noted that "*AD trials done prior to obligatory amyloid-PET imaging turned out to have up to ~25% of subjects that were amyloid-negative*" 20. Thus, the unsuitable choice of trial candidates may also be one of the factors contributing to the failure of some clinical candidates.

Aside from anti-amyloid agents, a study published in 2016 suggested that gamma oscillations may be a prospect for treatment of AD by reducing total amyloid levels via decreased amyloidogenesis and increased amyloid endocytosis by microglia, and the effects were not specific to one animal model 87. In addition, Sorrentino *et al*. reported that mitochondrial abnormalities also play a role in the pathogenesis of AD and that boosting mitochondrial function and proteostasis might decrease the formation of Aβ aggregates 88. Thus, mitochondrial proteostasis may also provide a new insight to amyloid-targeted therapies. Moreover, Ono reported that rosmarinic acid (RA) could inhibit Aβ40/42 oligomerization and decrease oligomer-induced synaptic toxicity 41.

## CONCLUSIONS

The discovery of amyloid plaques in the brain tissue of AD patients and subsequent findings concerning APP genes naturally led to the amyloid cascade hypothesis. Unsurprisingly, Aβ peptide plays an important role in the course of AD development. In the more than 20 years since the proposal of the original hypothesis, a substantial number of published reports have helped bolster research on AD and treatment strategies. Serious concerns have been raised about efficacy, however, yet new discoveries have been made. The amyloid cascade hypothesis, although still controversial, continues to help guide AD research. It is agreed that Aβ oligomers, instead of amyloid plaques, constitute the primary cause of toxicity, and Aβ42 seems to be more toxic than Aβ40, as the length of the peptide determines the toxicity 45. Aβ oligomers disrupt synaptic activity, although Aβ has now been shown not to be responsible for certain pathological effects of AD, as was previously presumed. An intermediate level of Aβ enhances presynaptic excitation whereas elevated or reduced levels depress synaptic function. Moreover, Aβ oligomers have opposite effects on excitatory and inhibitory synapses, and thus their impact on the neural circuitry varies depending on the structures of neuronal networks. A high level of Aβ-induced neuronal hyperexcitability, aberrant neuronal network activity, and dysfunction of slow oscillations can lead to impairment of learning and memory. Many attempts have been made to develop drugs that reduce the level of Aβ. In clinical trials, immunotherapy has been more successful by far than β/γ-secretase inhibitors. However, further studies are needed to improve cognitive outcomes in addition to removing Aβ plaques more efficiently. Nevertheless, AD is multifactorial disease, and a more integrated approach must be applied to increase treatment efficacy. Although immunotherapy holds promise, innovative approaches such as gamma oscillations and mitochondrial proteostasis, among others, have shown promising results. Further research may yield a more efficacious therapy. Notably, the discovery of the connection between amyloid plaques and tau aggregation indicates that future treatment of AD might not be based solely on the amyloid cascade hypothesis.

## Abbreviations

Aβ: amyloid β
AD: Alzheimer’s disease
APP: amyloid precursor protein
BBB: blood-brain barrier
FAD: familial AD
LDLR: low-density lipoprotein receptor
LTD: long-term depression
LTP: long-term potentiation
NMDA: N-methyl-D-aspartic acid
NMDAR: NMDA receptor
PrP^C^: cellular prion protein
